# Radiofrequency Exposure Levels from Mobile Phone Base Stations in Outdoor Environments and an Underground Shopping Mall in Japan

**DOI:** 10.3390/ijerph18158068

**Published:** 2021-07-30

**Authors:** Teruo Onishi, Miwa Ikuyo, Kazuhiro Tobita, Sen Liu, Masao Taki, Soichi Watanabe

**Affiliations:** National Institute of Information and Communications Technology, Tokyo 184-8795, Japan; m_ikuyo@nict.go.jp (M.I.); ka_tobita@nict.go.jp (K.T.); liusen@nict.go.jp (S.L.); m_taki@nict.go.jp (M.T.); wata@nict.go.jp (S.W.)

**Keywords:** EMF exposure levels, monitoring, mobile base stations, outdoor environment, underground shopping mall, Japan

## Abstract

Recent progress in wireless technologies has made human exposure to electromagnetic fields (EMFs) increasingly complex. The situation can increase public concerns related to possible health effects due to EMF exposure. Monitoring EMF exposure levels and characterizing them are indispensable for risk communications of human exposure to EMFs. From this background, a project on the acquisition, accumulation, and applications of EMF exposure monitoring data in Japan was started in 2019. One of the objectives of this project is to obtain a comprehensive picture of EMF exposure in actual daily lives. In 2019 and 2020, we measured the electric field (E-field) strength from mainly mobile phone base stations in the same areas as those in measurements conducted in 2006 and 2007 by the Ministry of Internal Affairs and Communications (MIC), Japan, and compared the data to investigate the time-course of the EMF environment. The number of measured points was 100 (10 × 10 grids) in an area of 1 km × 1 km in two urban and two suburban areas, and that in an underground shopping mall was 158. This large-scale study is the first in Japan. As a result, we found that the measured E-field strengths tended to be higher in 2019 and 2020 than those in 2006 and 2007, especially in the mall. However, the median ratios to the Japanese radio wave protection guideline values for urban areas and malls are lower than −40 dB.

## 1. Introduction

Owing to the practical application of the fifth-generation mobile communication system (5G), whose commercial services were launched in Japan in 2020, the Internet of Things (IoT), wireless power transfer (WPT), etc., are increasing the opportunities for exposure to electromagnetic fields (EMFs) in a wide range of frequencies from low- to high-frequency bands over various wireless devices. Data are insufficient to actually evaluate the levels of exposure from various EMF sources and to grasp the transition of wireless technologies in an individual’s various activities in daily life. Research on exposure level monitoring has already been conducted mainly in Europe and other countries [1,2,3,4,5,6,7,8,9,10,11,12,13,14,15,16,17,18,19,20]. In some countries, the exposure levels from mobile phone base stations and broadcasting transmission towers have been monitored and disclosed [1,2,3,4,5,6,7,8]. In 2012, a comparative international analysis of radiofrequency (RF) exposure surveys was reported, which included measurement results in 23 countries between 2000 and 2010 [1]. The paper mainly reported exposure levels from Global System for Mobile Communications (GSM) and Wideband Code Division Multiple Access (WCDMA) base stations. Joseph et al. also reported their measurement results in three European countries (Belgium, The Netherlands, and Sweden) [2]. Kim et al. reported exposure levels from mobile base stations and broadcast towers in Korea [3]. Joseph et al. also evaluated exposure levels for Long-Term Evolution (LTE), which is the fourth-generation mobile communication system (4G) [9]. Since a large area cannot be covered by fixed measurements, car mounted measurement methods were studied [10,11]. The disadvantage of this method is that the accuracy is less than that of a fixed measurement. Joseph et al. introduced an exposure assessment using drones [12]. It provides three-dimensional measurements at locations that are difficult to access. Another exposure measurement method is the use of a portable device that enables the measurement of the electric field (E-field) around the human body. Since the above measurements cannot constantly monitor an individual’s exposure, it is useful for us to use a portable device, especially for an epidemiological study. Sagar et al. reported the results of their measurements using portable devices conducted in Switzerland, Ethiopia, Nepal, South Africa, Australia, and the United States of America [13]. Another important point is to investigate the actual EMF level in the vicinity of a mobile phone, because they are usually used close to the head and the body. Thus, exposure levels from mobile phones were also evaluated in other countries [14,15,16]. Since it is difficult to evaluate exposure levels anywhere and anytime, some techniques to extrapolate and estimate using measurement or simulation data are proposed and studied [17,18,19,20,21]. The artificial neural network is a potential candidate to improve prediction accuracy. Recently, 5G services have been introduced in some countries. Measurement methods for exposure levels from 5G base stations and mobile phones have been studied [4,5,6,7,16]. Such research, however, has not been performed so far in Japan.

With the above as background, since 2019, we have been conducting research to mainly quantitatively determine the actual state of exposure to EMFs in daily life and to examine the appropriate manner of risk communication.

In this paper, we report the results of E-field measurements related to mobile phone base stations, which are one of the main sources of exposure in a general environment, in outdoor environments and an underground shopping mall in Japan in 2019 and 2020. The measurements were conducted in the same areas as those in measurements carried out by the Ministry of Internal Affairs and Communications (MIC), Japan, in 2006 and 2007 [22,23]. Although the present measurement conditions are not precisely the same as those in the previous measurements, it is useful to investigate the time-course of the EMF environment. A survey of time-courses in some countries before 2010 was reported in [1]. A critical point of our study is that it shows recent comparison results in the same areas of both outdoor and indoor environments.

## 2. Materials and Methods

In 2006, the E-field strength was measured at 100 points (10 × 10 grids) in an area of 1 km × 1 km in two urban and two suburban areas [22]. In addition, similar measurements were conducted at 158 points in an underground shopping mall in 2007 [23]. In this study, E-field strength measurements in the same areas (Urban areas—A and B and Suburban areas—A and B) and the same underground shopping mall as described in the previous reports [22,23] were conducted using a spectrum analyzer (SA) with a three-axis isotropic E-field probe (SRM-3006, Narda S.T.S. GmbH) in 2019 and 2020. Either the E-field, magnetic field, or incident power density can be used for RF exposure assessment. We selected the E-field so that the obtained results could be directly compared with past results. The measurements in the mall were conducted during the daytime (9:00–16:30) and night-time (17:00–22:30), similar to the previous measurements, whereas the measurements in the outdoor environment were carried out from 8:30 to 17:30. Note that the shopping mall is directly connected to one of Japan’s major stations and is usually crowded day and night. However, there were slightly fewer people than usual due to COVID-19 when the measurements were performed. The frequency bands we mainly measured for E-field strengths are 700, 800, 900, 1500, 1700, 2000, 2500, and 3500 MHz, which were allocated for mobile phone systems in Japan at that time. Measurements were also carried out in the industrial, scientific, and medical (ISM) bands used for wireless local area network (LAN) and 1900 MHz band used for personal handy-phone system (PHS). Note that 5G was not considered for the measurements in 2019 and 2020 because 5G services were not available yet in Japan at the time when the outdoor measurements were conducted, and 5G services were not available in the mall in 2020. The resolution bandwidth (*RBW*) and video bandwidth (VBW) of the spectrum analyzer were set to 1 MHz [24] and 100 kHz, respectively. From the viewpoint of radio wave protection, an average E-field strength over 6 min is required instead of an instantaneous value [25]. It was reported that the E-field strength averaged over 1 min only varied within 0.5 dB from that over 6 min [26]. Similar results were obtained by our measurements as shown in Table 1. Therefore, 1 min was used to obtain the average E-field strength to reduce measurement time. First, the probe was swept in the vertical direction between 10 and 200 cm at 10 cm intervals above the ground or floor at each measurement point to determine the height at which the E-field strength was maximum according to [27,28]. The probe was fixed to a probe holder made of fiber reinforced plastics (FRPs) and connected to the SA with a dedicated cable, so as to reduce the effect of the human body (i.e., the operator) during measurements. Then, the E-field strength was measured, and the average over 1 min at the height was obtained. The square root of the sum of the squares of the measured field strengths (*E_int_*) was calculated by Equation (1) [29] to obtain the field strengths for each desired frequency band. Δ*f* is a frequency resolution (Hz), and *f*_1_ and *f*_1_ + (*n* − 1) × Δ*f* are the lowest and highest frequencies in a desired frequency bandwidth, respectively.

(1) $$E_{int}=\sqrt{\frac{\Delta f}{1.0552RBW}\overset{n-1}{\underset{k=0}{\sum }}{\left(E\left(f_{1}+k\Delta f\right)\right)}^{2}}$$

## 3. Results

**Figure 1:**
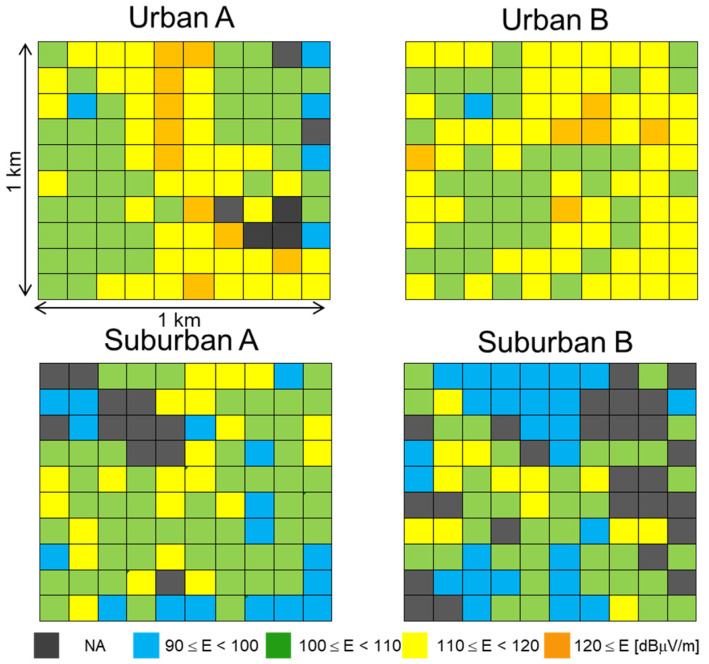
E-field strength in all frequency bands in the outdoor environments.

**Figure 2:**
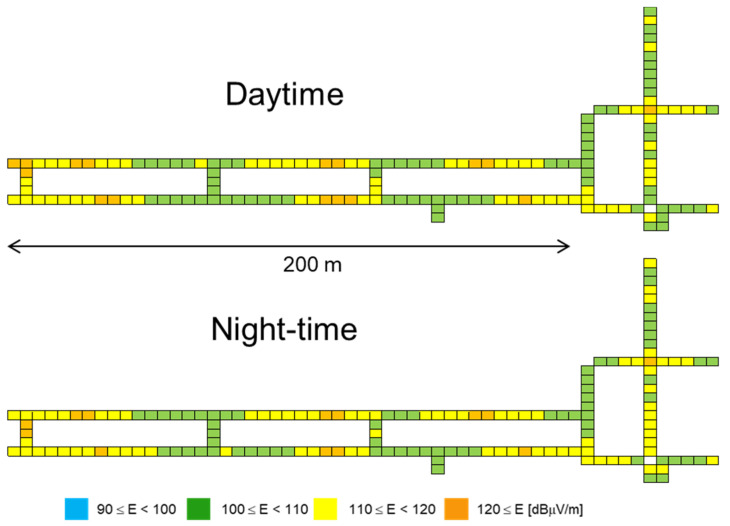
E-field strength in all frequency bands in the underground shopping mall.

Examples of the measurement results in the outdoor environments and the underground shopping mall are shown in Figure 1 and Figure 2 as a color map, respectively. The total E-field strength is shown as the effective value obtained by integrating (the square root of the sum of squares) them all over the desired frequency bands. The NA (black) indicates that the measurement was not possible because the area was private land, a forest, or another type. The E-field strength in the urban areas including the mall tends to be larger than that in the suburban areas.

**Figure 3:**
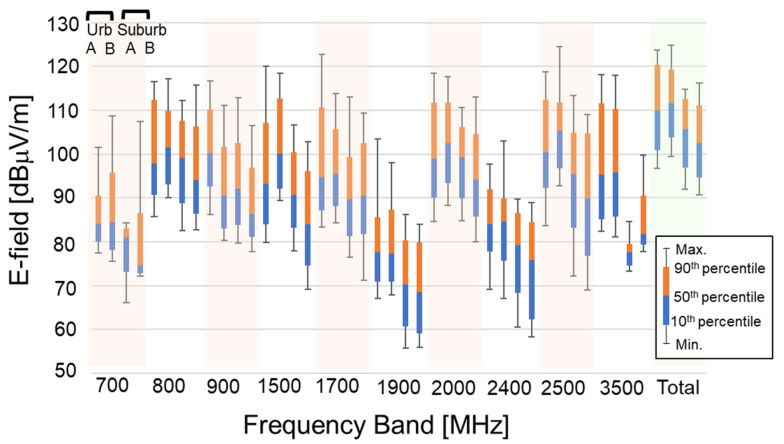
Statistical distribution of E-field strength in each frequency band and total frequency bands in the outdoor environments.

Figure 3 shows the maximum; the 90th, 50th (median), and 10th percentiles; and the minimum E-field strengths for each frequency band in the outdoor environments. As described in Section 2, measurements at frequency bands for 5G were not carried out because 5G services were not yet available in 2019. The total median E-field strengths in the urban areas were about 7 dB larger than those in the suburban areas, as shown in Figure 1. The E-field strengths in the urban areas also tended to be larger than those in the suburban areas in the individual frequency bands. In particular, the regional differences between the urban and suburban areas were remarkable except for the 800, 900, and 2000 MHz bands. The E-field strengths in the suburban areas were about 20 dB smaller than those in the urban areas in the 3500 MHz band. Furthermore, the E-field strengths in the 700 MHz band were about 10 to 20 dB lower than those in the other mobile phone bands. Since these two frequency bands have been allocated recently, it seems that the installation of base station equipment for these bands is in progress. It is also obvious that the E-field strengths in the 1900 and 2400 MHz bands were lower as well.

**Figure 4:**
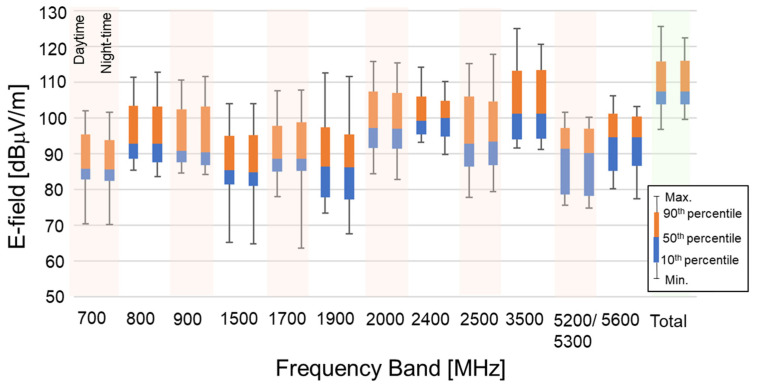
Statistical distribution of E-field strength in each frequency band and total frequency bands in the mall.

Figure 4 also shows the E-field strength distribution in each frequency band in the mall. It is clear that the differences in E-field strength between daytime and night-time are marginal. The E-field strengths in the 2000 and 3500 MHz bands are larger than those in other bands. For example, about 5 and 10 dB smaller median values were observed in the 800 and 900 MHz bands than in the 2000 and 3500 MHz bands, respectively. The median E-field strength in the 2400 MHz ISM band is the same as that in the 2000 MHz band, whereas the median E-field strengths in the 5000 MHz ISM bands are 5–9 dB smaller than in the 2000 and 2400 MHz bands. One of the reasons may be that the antennas of wireless LAN equipment (access point; AP) are densely located on the ceiling, and the wireless LAN is frequently used by passers-by and people in coffee shops, restaurant, and stores.

## 4. Discussion

### 4.1. Exposure Levels Compared with Limits

Measurement results were compared with limits in general environments in the radio wave protection guidelines in Japan, as shown in Table 2. The E-field strength in the radio wave protection guidelines is proportional to the square root of the frequency in between 300 MHz and 1.5 GHz and is constant at 61.4 V/m (155.76 dBμV/m) at frequencies above 1.5 GHz [25]. The minimum E-field strength in the guidelines at the target frequency of this measurement is 44.1 V/m (152.89 dBμV/m). Therefore, the ratio to the guideline value in each frequency band and the sum of squares were calculated. A measurement of 0 dB means the same level as the limits. As shown in Table 2, the maximum ratios in the urban areas and the mall are about 10 times higher than those in the suburban areas; however, they were lower than −20 dB from the level of the Japanese guidelines. If we focus on the median value, the ratio is approximately on the order of the −40 dB from the limit.

### 4.2. Comparison of Present Data with Previous Data

The measurement results in 2019 and 2020 were compared with those in 2006 and 2007. The numbers of mobile phone base stations in Japan were about 475,000 (excluding femto-cell base stations) in 2020 and 140,000 in 2006 [30]. This means that the current number of the base stations is about 3.4 times more. Table 3 summarizes the downlink frequency bands of mobile phone base stations and the generations of mobile phone systems. Note that the PHS and mobile phone systems operating in the 2500 and 3500 MHz bands use the time division duplex (TDD). In 2006, the second-generation (2G) and third-generation (3G) mobile phone systems and PHS were the only systems in operation. The available frequency bands at that time for measurements were the 800, 1500, 1700, and 2000 MHz bands [22,23]. On the other hand, it can be seen that the total bandwidth increased 3.1-fold, and frequencies higher than the 2000 MHz band were newly used at the time we conducted the measurement. Other important conditions, such the transmission power and antenna gain of the base station, which are directly related to the E-field strength, are unknown because these are not disclosed as far as we know.

**Figure 5:**
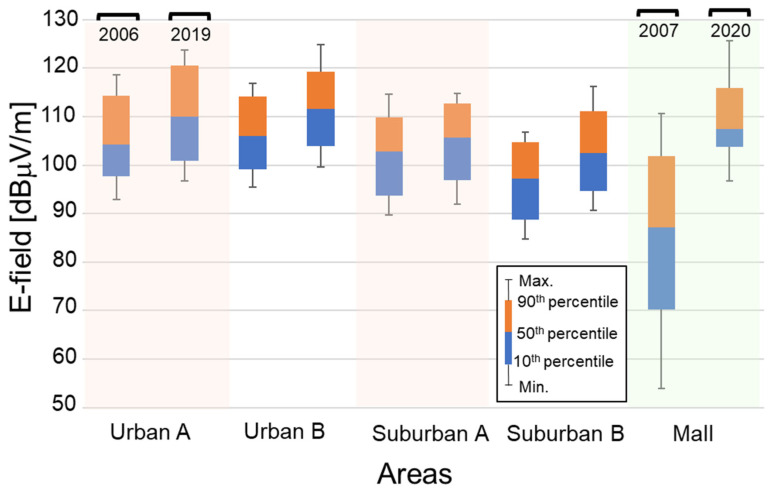
E-field strength distributions in all frequency bands.

**Figure 6:**
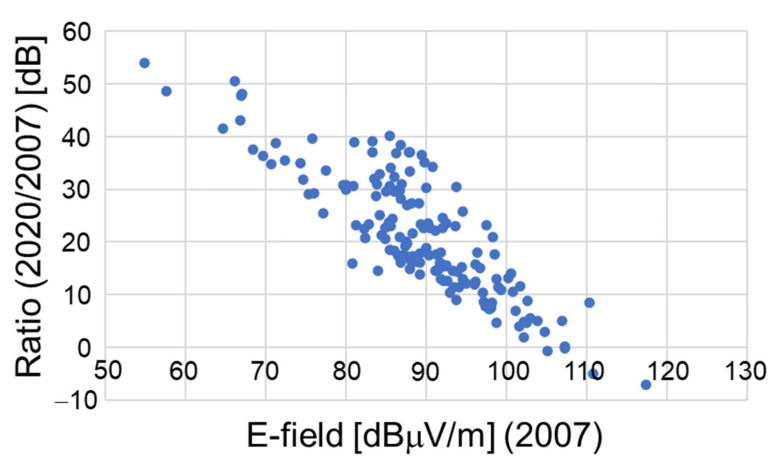
Ratio of E-field strengths in 2020 to those in 2007 point by point in all frequency bands.

To compare the past and present results, the ratio of the maximum to the time-averaged E-field strengths was evaluated at each frequency band because the past measurements were not conducted based on time-averaged values but on the maximum hold. Table 4 summarizes the ratios in the outdoor environments and the underground mall. The ratios are almost the same even though the environments were different. The comparison was performed using the E-field strengths obtained by dividing the E-field strengths in 2006 and 2007 with the ratios. Figure 5 shows the statistical distribution of E-field strengths in all frequency bands in both areas in 2019 and 2020 compared with those in 2006 and 2007. It is validated with statistical significances by the *t*-test (*p* < 0.001) that the E-field strengths in the outdoor environment are increased. It is also clear that the median E-field strengths in the mall increased from 2007 to 2020 at the ratio of 20.3 dB. Note that similar tendencies are obtained even though only frequency bands allocated in the past are considered. It is presumed that the increase in E-field strength may be related to the increase in the number of base stations in 2020, which is 3.4 times more, as well as the frequency bands. In addition, E-field strengths in the mall are compared point by point as shown in Figure 6. The interesting observation is that the ratio increased as the E-field strengths in 2007 decreased.

### 4.3. Comparison with Other Countries

A comparative international analysis among 23 countries was performed by Rowley and Joyner in 2012 [1], which included part of the Japanese results. The data for the analysis were measured between 2000 and 2010 depending on countries. The average power density was 0.0567 μW/cm^2^, which is equal to 113.3 dBμV/m for over 21 countries. Additionally, time-course results among five countries were analyzed, which showed that exposure levels were not markedly different. Our estimated E-field strengths in outdoor environments reported in 2006 [21], as shown in Figure 5, are approximately 10 dB smaller than those reported in [1]. This tendency is similar to that described in [1]. However, our results indicate that E-field strengths in 2019 and 2020 increased compared with the past results, contrary to [1]. Ofcom, UK, reported its measurement results using the SRM-3006 equipment [6], which is the same equipment used in our study. Measurements were carried out including the 5G base stations in 22 locations across England, Scotland, Wales, and Northern Ireland. The highest exposure level in measurements including the 5G system was approximately 1.5% (−18.2 dB) of the reference level of the International Commission on Non-Ionizing Radiation Protection (ICNIRP). Since the highest level for only 5G is 0.039% (−34.1 dB), other systems, i.e., 2G, 3G, and 4G mobile base stations, are the predominant radiation sources in the measurements. The exposure levels in the 22 locations were between 0.04% (−34 dB) and 1.5% (−18.2 dB). Our results in the outdoor environments were −29.2 dB and −28.1 dB in the urban areas A and B and −38.2 dB and −36.8 dB in the suburban areas A and B, respectively, as shown in Table 2. The values we obtained in outdoor environments are slightly smaller than those in the UK.

## 5. Conclusions

The results of E-field strength measurements in mobile phone base stations, which are one of the main sources of EMF exposure in the general environment, namely in outdoor environments and an underground shopping mall in Japan in 2019 and 2020, have been presented. The measurements were conducted in the same areas as those conducted by MIC, Japan, in 2006 and 2007.

As a result, we found that the total median E-field strengths in the urban areas are about 7 dB larger than those in the suburban areas. The E-field strengths in the urban areas also tend to be larger than those in the suburban areas in individual frequency bands. For the shopping mall, it is clear that the differences in E-field strengths between daytime and night-time are marginal. The E-field strengths in the 2000 and 3500 MHz bands are larger than those in other bands. The E-field strengths in the 2400 MHz ISM band are the same as those in the 2000 MHz band, whereas the median E-field strengths in the 5000 MHz ISM bands are 5–9 dB smaller.

The measured results were compared with the Japanese radio wave protection guidelines. As a result, we found amounts lower than the limits by −20 dB. If the median E-field strength is focused on, the ratio is approximately on the order of −40 dB with respect to the limit. Compared with the previous data in 2006 and 2007, it is clear that the E-field strengths in both outdoor environments and the mall increased, especially in the mall, whose ratio of the E-field strength in 2007 to that in 2020 is 20.3 dB. Although the present measurement conditions are not precisely the same as those in the previous measurements, it is useful to investigate the time-course of EMFs in various environments. Additionally, the E-field strengths we obtained in outdoor environments are slightly smaller than those in another country.

Since our measurements were carried out before the start of 5G commercial service, the exposure level at the frequency band used in 5G was not included. However, measurements of exposure levels for 5G and new radio waves used will be continued. Due to the limited time and space of the measurements in this study, exposure level data under various conditions of daily life will be accumulated in the future.

## Figures and Tables

**Table 1:** Maximum deviations of E-field strength averaged over 1 min from that averaged over 6 min.

Frequency Range [MHz]	700	1500	2100
Maximum deviation [dB]	0.5	0.6	0.5

**Table 2:** Ratio of E-field strength to values in the Japanese guidelines.

Item	Ratio [dB]					
	Urban-A	Urban-B	Suburban-A	Suburban-B	Mall (Day)	Mall (Night)
Maximum	−29.2	−28.1	−38.2	−36.8	−30.1	−33.1
Median	−42.9	−41.4	−47.3	−50.4	−48.0	−48.2

**Table 3:** Frequency and bandwidth of mobile phone systems except for 5G.

Frequency Band (MHz)	2006/2007		2019/2020	
	Bandwidth (MHz)	Generation	Bandwidth (MHz)	Generation
700	-	-	30	4G
800	44	2G/3G	30	3G/4G
900	-	-	15	4G
1500	27	2G	35	4G
1700	20	3G	75	4G
1900	32.6	PHS	31	PHS/TD-LTE
2000	60	3G	60	3G/4G
2500	-	-	100	BWA
3500	-	-	200	4G
Total	183.6	-	576	-

**Table 4:** Ratio of the maximum E-field strength to the time-averaged E-field strength.

Location	Ratio [dB] in Each Frequency Band			
	800 [MHz]	1500	1700	2100
Outdoor environments	7.0	8.0	8.4	8.1
Underground mall	7.5	8.2	9.4	8.3
